# Applying the Taguchi Method to Improve Key Parameters of Extrusion Vacuum-Forming Quality

**DOI:** 10.3390/polym16081113

**Published:** 2024-04-16

**Authors:** Dyi-Cheng Chen, Der-Fa Chen, Shih-Ming Huang

**Affiliations:** 1 Department of Industrial Education and Technology, National Changhua University of Education, No. 1, Jin-De Road, Changhua City 500, Taiwan; dfchen@cc.ncue.edu.tw; 2 Department of Mechanical Engineering, Wu Feng University, No. 117, Section 2, Jianguo Road, Minxiong 621303, Taiwan

**Keywords:** extrusion molding, Delphi technique, Taguchi method, quality control

## Abstract

This research investigates the control of thickness and weight in plastic extrusion vacuum-thermoforming products to identify optimal key parameters for cost reduction and energy savings. The initial step involves identifying crucial influencing factors. In this step, the Delphi technique was employed through a questionnaire administered to a panel of expert scholars to ensure minimal error and maximal reliability in determining key influencing factors. Consensus was sought to establish appropriateness and consistency. Subsequently, the Taguchi method was applied for quality design and planning of the extrusion vacuum-forming process. The experimental design parameters were selected using an L_18_ (2^1^ × 3^7^) orthogonal array, and the desired quality characteristics were determined. Comparative analysis of quantitative production data from two consecutive experiments was conducted, and based on F-values and contribution analysis, the combination of control factors maximizing the Signal-to-Noise (S/N) ratio was identified. The objective is to seek optimal parameters for improving the quality of the plastic polypropylene (PP cup lid) manufacturing process, reducing process variability, and identifying the most robust production conditions. Through multiple actual production prediction experiments, it was determined that five control factors, “polypropylene new material ratio,” “T-die lips adjustment thickness”, “mirror wheel temperature stability”, “molding vacuum pressure time”, and “forming mold area design”, contribute to the maximization of the S/N ratio, i.e., minimizing variability. Statistical validation confirms a significant improvement in product quality and weight control. Noteworthily, the quality control model and experimental design parameters established in this study are also applicable to other plastic products and bio-based materials, such as PET, HIPS, and biodegradable PLA lids with added calcium carbonate. The results of the experimental production demonstrate its ability to consistently control product weight within the range of 3.4 ± 0.1 g, approaching the specified tolerance limits. This capability results in approximately 2.6% cost savings in product weight, contributing significantly to achieving a company’s KPI goals for environmental conservation, energy efficiency, and operational cost reduction. Therefore, the findings of this study represent a substantial and tangible contribution.

## 1. Introduction

In light of the investigation conducted by the non-governmental environmental organization Greenpeace International [1], the surge in the quantity of single-use plastic products [2] during the COVID-19 pandemic proves challenging to quantify. “Plastic reduction” [3] has become the trend, and the consensus of environmental protection in various countries around the world [4] has gained momentum, particularly in relation to food-grade utensils and beverage packaging containers [5]. In the rational intersection of environmentalism and everyday life, a genuine reduction in pollution necessitates a scientific approach that targets the control of sources [6], thereby enhancing production efficiency, quality improvement, and rational reduction in product weight.

**Figure 1:**
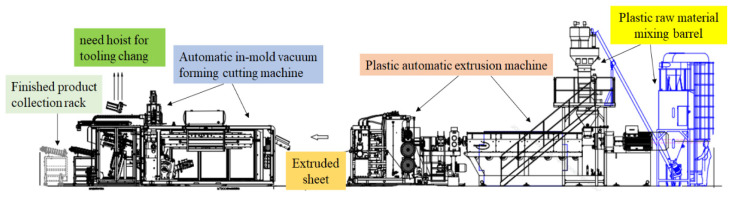
Plastic extrusion molding production process [13].

Polypropylene, known for its versatility [7], finds application in food packaging [8], microwave-resistant containers, piping, and automotive components. Examples include beverage cup lids, lunchboxes, frozen liner boxes, and food packaging materials such as plastic cutlery. Extrusion molding [9], which utilizes an extruder [10], involves thoroughly mixing and heating plastic fiber or sheet-like polymer raw materials to form sheets. These molten sheets or film materials are then continuously heated and vacuum-formed through aluminum alloy molds [11] or shaping modules [12], followed by cooling and cutting to produce various products. The process [13] is illustrated in Figure 1.

## 2. Literature

This study is based on material characteristic inspection reports provided by various brand companies in the Asian region [14] and practical experiences accumulated over 25 years of production management in the manufacturing of polypropylene packaging containers by Company H [15]. These practical experiences include technological expertise and quality control inspection statistics. In addition, the study synthesizes and organizes key considerations for the incoming quality control (IQC) of raw materials in the production of plastic polypropylene food packaging products. This research aims to offer valuable insights to procurement personnel within enterprises, aiding them in selecting optimal reference values and reference value ranges for material characteristics, as presented in Table 1.

## 3. Research Method and Analysis Experiment

### 3.1. Analysis Based on Three Iterations of the Delphi Technique

**Figure 2:**
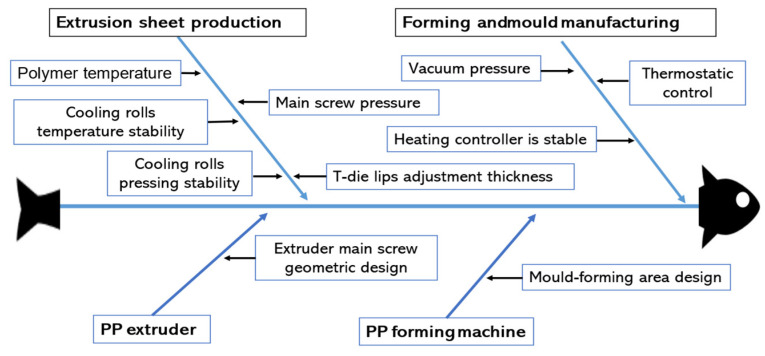
10 key quality factors for polypropylene extrusion molding [19].

Building upon three iterations of the Delphi technique involving expert scholars [16], the analysis results revealed the primary facets of the four key dimensions influencing the quality of polypropylene extrusion molding. The quality control resolution process is illustrated with a fishbone diagram [17,18] in Figure 2. From this analysis, we determined the ten most critical success factor elements.

Our study conducted three Delphi structured questionnaire interviews. Statistical results of the third Delphi questionnaire revealed that (1) the high fitness selection criteria was employed with the mean of ≥4.2, (2) the consistency selection standard deviation was ≤0.5, (3) the consensus of the K-S test has reached consistency, and (4) the subitems of progressive significance reached the significance level of ** *p* < 0.01. The items would be kept only when the above four requirements were met. Based on the final statistical analysis results of the third Delphi research questionnaire, the subitems of ten key control factors in production quality were obtained, as shown in Table 2 [19].

### 3.2. Analysis of Variance (ANOVA)

The analysis of variance (ANOVA) [20] serves as one of the statistical analysis tools for the Taguchi experimental design data. It employs mathematical formulas for the sum of squares to quantitatively assess the deviation values of each control factor’s effect on the overall experimental mean effect. The calculation formula is presented in Table 3.

### 3.3. Taguchi Method: Optimization of the Key Factor Parameter Experiment [22]

**Figure 3:**
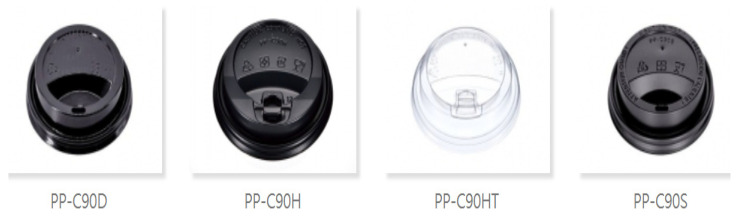
Various plastic polypropylene coffee cup lids of 90 mm diameter [15].

The experimental production samples mainly included “PP coffee cup lids”, as illustrated in Figure 3. This study optimized the quality characteristic of the stability of the “product weight standard error”, as depicted in the diagram. The primary objective of this research was to maintain the standard error value within the range of 3.4 ± 0.1 g, making the stability of this standard error the key quality characteristic. In other words, the closer the quality characteristic of the product weight is to the target value of 3.4 g, the more favorable the outcome. This was confirmed as the “desired characteristic” for the optimization of the quality characteristic.

Summarizing the factors influencing the quality characteristics of polypropylene extruded sheets and vacuum-formed products, we identified the following factors: “A. polypropylene new material ratio”; “B. extrusion screw pressure speed”; “C. polymer temperature”; “D. T-die lips adjustment thickness”; “E. mirror wheel temperature stability”; “molding heating thermostatic control”; “G. molding vacuum pressure time”; and “H. forming mold area design”. The experimental design selected an orthogonal array [23] based on eight control factors. Among these, 1 factor has two levels (21), and 7 factors have three levels each (37), forming a predominantly “mixed level” “design. The chosen experimental quality characteristic orthogonal array is denoted as L_18_ (2^1^ × 3^7^), as illustrated in Table 4.

### 3.4. Experimental Procedure of the Study

**Figure 4:**
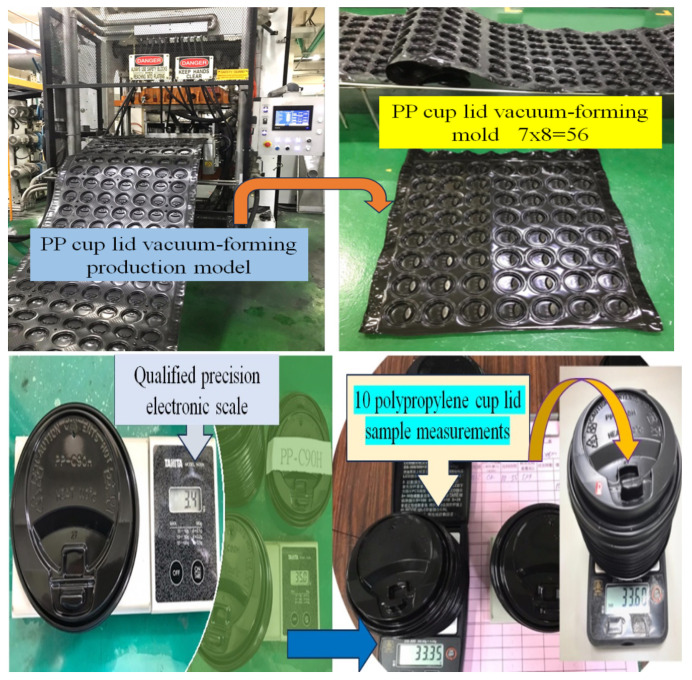
Weighing method of experimental PP cup lid samples.

In accordance with an orthogonal array L_18_ (2^1^ × 3^7^) revised by engineers [24], the experimental setup involved the utilization of 0.55 mm polypropylene extruded sheets for molding production experiments. Following the original design parameters, experiments were conducted at L Factory, and the products were subjected to inspection and quantification. A total of 18 experimental sets were conducted, with the factory packaging personnel preselecting qualified products. For each row, 7 lid samples were randomly selected and sampled, and 10 lid samples from the same column were weighed using an electronic scale. The experimental records are illustrated in Figure 4.

Setting the minimum baseline error to ±0.05 g, an average weight rounding to the nearest decimal was adopted for each weight measurement. The weights for each experimental cup lid sample—denoted as P1 to P7 (unit: g)—were recorded in adherence to the predetermined specifications outlined in orthogonal array L_18_ and the numerical values corresponding to control factors A to H, as presented in Table 5.

**Figure 5:**
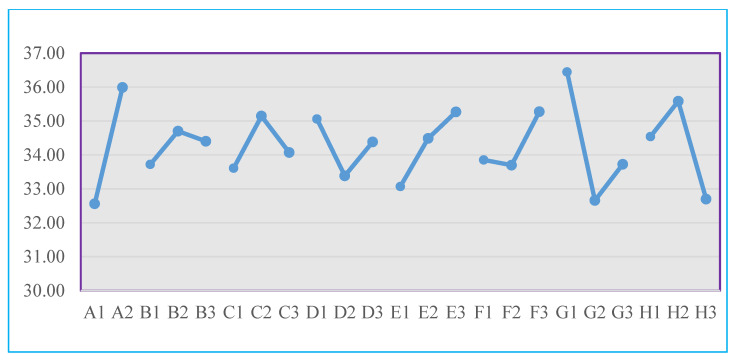
Factor response chart of the S/N ratio in each group of experiments.

The factor effects in the Taguchi method [25] refer to the magnitude of the impact of variations in control factors on Signal-to-Noise (S/N) ratio or quality characteristics. The calculations of the factor response table for the S/N ratio were conducted for each experimental set from factors A to H. The statistical data for the factor response table and factor response plot are presented in Table 6 and Figure 5.

Based on Table 4 and Figure 4, insights gleaned from the data of the factor response tables and plots for the S/N ratio in each experimental set reveal that control factor G at Level 1 demonstrates the highest significance, representing the optimal S/N ratio. Further, the analysis of the factor response plots for the S/N ratio in each experimental set shows that the optimal combination of factor levels is identified as A2, B2, C2, D1, E3, F3, G1, and H2, as outlined in Table 7.

**Figure 6:**
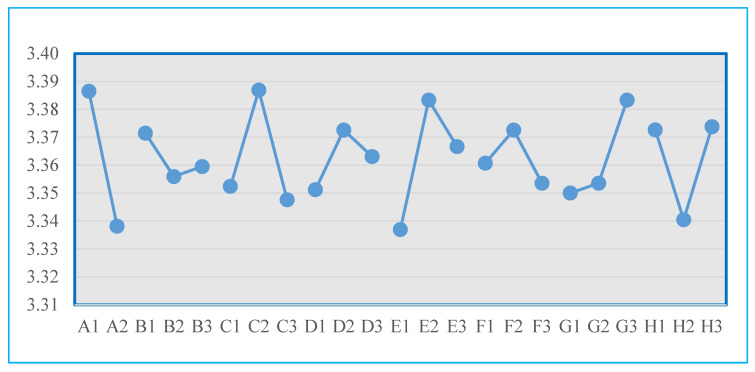
Factor response diagram for each group of experimental quality characteristics.

The quality objective of the experiment is to achieve an average value of the quality characteristic at the target value of 3.4 ± 0.1 g. Therefore, following the same procedure based on the S/N ratio, a response analysis was conducted for the quality characteristic. Utilizing the “one-half criterion” method [26], factors significantly influencing the characteristic were selected, resulting in the identification of the top five important factors. The remaining three were deemed unimportant. Notably, factors A, C, and E were observed to influence the quality characteristic (product weight), whereas factors B and F had minimal impact on the product weight. The calculations were rounded to the nearest decimal. For the D factor, slight differences in the range section were observed for better-ranking comparison. The data from the factor response tables and plots for the analysis of the quality characteristics in each experimental set are presented in Table 8 and Figure 6.

### 3.5. Analysis of Variance (ANOVA)

Analysis of variance [20] was employed to assess errors in the experiment. Generally, when there are only two factors, *t*-tests [27] can be used; however, when there are more than two factors, ANOVA is more appropriate. In the Taguchi method, interactions among control factors are considered potential experimental errors. The variance within groups primarily arises from external noise [28]. If the factor effects exceed the experimental error, the factor effects are deemed significant. In understanding the impact of each control factor parameter on product weight and quality, several variance calculations were conducted, including average variance and square (CF), total sum of squares (SST), sum of squares for variable ij (SSj), error sum of squares (Se), variance for variable j (Vj), error variance (Ve), and variance ratio for variable j (Fj). Here, n is the sample size for each level of a factor, and degrees of freedom (DOF) [29] are calculated as n minus 1 (n−1) for each factor level. Based on the aforementioned formulas, preliminary statistical data for variance analysis were calculated, as presented in Table 9.

The ratio of the *F*-distribution, referred to as the *F*-value [30], is computed to explain the ratio of two variances. A higher *F*-value indicates a smaller likelihood that the control factor comes from the same sample space, signifying greater influence and importance of that control factor. In the preliminary analysis of variance statistical data (Table 7), an *F*-value smaller than 1 indicates a small impact of the control factor, and an *F*-value greater than 2 suggests significant importance. The “*” symbol denotes a significant control factor. The *F*-values corroborate the significance ranking of control factors, which are A, E, C, H, and G, according to the statistical data from the factor response table (Table 5) for each experimental set.

### 3.6. Experimental Confirmation

The research conclusions drawn from the data analysis in Table 7 should be validated. For effective estimation of each observed value, the verification of the experiment involves calculating confidence intervals [31] below the confidence level. Based on the variance analysis, the confidence intervals for the predicted mean values of the optimal parameter combination should be set to at least 90%. This ensures that the F-values and their corresponding levels for the selected significant factors are appropriate and meaningful. Additionally, the contribution percentages (P) of each significant control factor were computed. The variance analysis data after consolidating errors were summarized, as presented in Table 10.

In the process of confirming the optimal manufacturing procedure, it is essential to assume that the effects of control factors are independent. When the levels of control factors change, the Signal-to-Noise (*S*/*N*) ratio increases, and there is mutual influence among the factors. For instance, when the setting values of the factors at the A2 level change with the B2 level factors, it is considered an interaction between two factors. Therefore, control factor effects can only be considered independent when there is no interaction between control factors. Thus, the conclusion of the optimal control factor level combination in this study—A2, B2, C2, D1, E3, F3, G1, and H2 (Table 5)—is reliable and less prone to suspicion. The ability to consider control factor effects independently implies that the effects can be calculated cumulatively. For example, transitioning from the original process (A2, B2, C2, D2, E2, F2, G2, H2) to the optimal process (A2, B2, C2, D1, E3, F3, G1, H2) results in an increase in the S/N ratio. Since the effects of “non-significant control factors” are statistically insignificant, the increase in the *S*/*N* ratio in studying the optimal process is only considered for the effects of significant control factors (A2, D1, E3, G1, H2). The formula is calculated as follows.

$$\begin{array}{cc} \Delta S/N & =E_{A}^{2\to 2}+E_{D}^{2\to 1}+E_{E}^{2\to 3}+E_{G}^{2\to 1}+E_{H}^{2\to 2}\\ & =0+1.67+0.78+3.79+0\\ & =6.24 \end{array}$$

(1) The original process *S*/*N* ratio formula calculation is predicted.
  $$\begin{array}{cc} \eta_{original} & =\bar{\eta }+\left({\bar{\eta }}_{A2}-\bar{\eta }\right)+\left({\bar{\eta }}_{D2}-\bar{\eta }\right)+\left({\bar{\eta }}_{E2}-\bar{\eta }\right)+\left({\bar{\eta }}_{G2}-\bar{\eta }\right)+\left({\bar{\eta }}_{H2}-\bar{\eta }\right)\\ & =34.3+(36-34.3)+(33.4-34.3)+(34.5-34.3)+(32.7-34.3)+(35.6-34.3)\\ & =35 \end{array}$$
(2) The *S*/*N* ratio formula under the best combination is calculated.
  $$\begin{array}{cc} \eta_{optimal} & =\bar{\eta }+\left({\bar{\eta }}_{A2}-\bar{\eta }\right)+\left({\bar{\eta }}_{D1}-\bar{\eta }\right)+\left({\bar{\eta }}_{E3}-\bar{\eta }\right)+\left({\bar{\eta }}_{G1}-\bar{\eta }\right)+\left({\bar{\eta }}_{H2}-\bar{\eta }\right)\\ & =34.3+(36-34.3)+(35.1-34.3)+(35.3-34.3)+(36.4-34.3)+(35.6-34.3)\\ & =41.2 \end{array}$$
(3) From the original process to the optimal process, the calculation formula for the increase in *S*/*N* ratio is:
  ΔS/N=41.2−35=6.2

Although there is a discrepancy of ±0.04 when comparing the calculated increase in the Signal-to-Noise (*S*/*N*) ratio (6.2) with the original value (6.24), it falls within the reasonable error calculation range. The statistics employ rounding for precision. Therefore, the verified increase in the *S*/*N* ratio for the experimental control factors in the original and optimal designs should be considered as 6.2. The impact values (effect, dB) [32] of the significant control factors and the data predicted by the additive model [33] are presented in Table 11.

#### Experimental Comparison after Process Improvement [34]

Based on the predicted results of the optimal design model, a comparative production experiment was conducted by adjusting parameters according to the optimal levels of the five critical factors (A2, D1, E3, G1, H2). While the levels of the A2 and H2 factors remained unchanged, the D factor (T-die lips adjustment thickness) was adjusted from 0.56 mm to 0.53 mm, the E factor (mirror wheel temperature stability) was adjusted from 30 °C to 35 °C, and the G factor (molding vacuum pressure time) was adjusted from 4.2 s to 4 s. The optimized thickness of the extruded sheet was also adjusted from 0.55 mm to 0.52 mm. Further, the predicted average weight of the vacuum-formed PP cup lid was originally around 3.5 ± 0.1 g; the adjustments aimed to bring it closer to the target value set by the Taguchi method—that is, 3.4 ± 0.1 g.

The experimental study focused on the extrusion molding of PP-C90 cup lids with the primary goal of controlling product weight. The Taguchi method was applied to identify the optimal process design for achieving the ideal functional target characteristic [35], with the aim of stabilizing the error value within 3.4 ± 0.1 g. Considering key performance indicators (KPIs) and operational cost profitability [36], the predicted quality control setting assumed that the weight of PP-C90 cup lids could be stably controlled within ± the specified error. Approaching 3.3 g was deemed the optimal process design without adopting the “smaller-the-better” characteristic [37] as the research design goal. This method was applied to avoid potential quality instability and customer complaints caused by excessively light product weight. The comparison between the experimental confirmation values and the predicted values of the optimal design is presented in Table 12. The specific research results on the important key aspects of Delphi methods [19] and the optimal parameters of the Taguchi method are explained in Table 13.

## 4. Conclusions

This study investigates the key factors for quality control in polypropylene extrusion molding. The ten key factors retained in the questionnaire were the consensus of 13 scholars and experts who considered these subitems the most critical factors in extrusion molding quality. The research framework is based on the experiential knowledge of experienced technical engineers and substantiated by a comprehensive review of international academic literature. Using professional machinery manufacturing and polypropylene production, actual empirical insights and data accumulation were repeatedly checked through experiments. The Taguchi and the Delphi methods were applied to identify ten crucial control factors. In addition, the L_18_(2^1^ × 3^7^) experimental orthogonal array was selected, and the desired characteristic was chosen as the research objective, making this a more innovative and practically executable research method.

1. Through repeated experiments, the study granted a deeper understanding of the correlation between quality and product weight in actual production. By identifying specific optimal design parameters, it was possible to reduce process variability and discover more robust production conditions. This approach facilitated the stabilization of product weight within the range of 3.4 ± 0.1 g and the determination of a robust design for mass production.

2. We recommend that manufacturing production managers refer to the process control methods and optimal design parameters of this study and also remind developers of special-purpose machinery to innovate and improve the direction. For example: an important key factor in reducing costs is to increase the B factor of the extrusion screw. The higher the pressure speed, that is, the greater the output of the extruder, the lower the production cost. In addition, the lower the ratio of new raw materials, the lower the production cost. However, the production of food-grade polypropylene packaging containers is based on the basic principle of safety and hygiene. Under the circumstances, it is required to invest new materials and non-landed recycled materials. The production cost of purchasing new materials and recycled materials is the same. Therefore, when the A factor is adjusted to increase the proportion of new materials from 50% to 60%, the increase in the relative molecular weight mixing ratio will improve the quality stability of the product, and the reduction in process variability can better control and reduce the quality error value.

3. The results of the two sets of experiments showed an average weight of 3.32 g. Compared to the original design value of 3.41 g, the results represent a reduction of 0.09 g. This reduction translates to approximately 2.6% cost savings in production weight. Taking an extrusion molding line experiment machine from Company H as an example—with a stable polypropylene extrusion output of approximately 280 kg/h, an 80% start-up rate, continuous production for 20 h/day, and 20 working days/month, assuming a high defect rate of 2%—the total production of sheet materials is estimated to be 87.8 tons/month. With a stable production weight reduction of 2.6%, the monthly savings in polypropylene raw material expenditure for cup lid production (excluding recycled material) are approximately 1.37 tons based on the optimal process design parameters from the research conclusion.

4. The calculated data from the research experiments demonstrate significant effectiveness in controlling the weight of extruded sheets and vacuum-formed products. Furthermore, the identified key factors and control factor parameters in the conclusion provide an effective method for saving raw production materials and assisting companies in reducing costs.

## Figures and Tables

**Table 1:** Optimal reference value ranges for material characteristics.

Selection of Reference Values for Raw Material Properties			
Typical Property	Test Method	Unit	Reference Value Range
Melt flow rate (230 °C, 2.16 kg)	ASTM D1238	g/10 min	1.6 ± 0.2
Density	ASTM D792	g/cm^3^	0.901 ± 0.001
Tensile strength at yield	ASTM D638	kg/cm^2^	370 ± 10
Flexural modulus	ASTM D790	kg/cm^2^	15,500 ± 1000
Rockwell hardness	ASTM D785	R scale	100 ± 5
23 °C Izod impact strength, notched 23 °C	ASTM D256	kg-cm/cm	5.0 ± 0.5
Deflection temperature (4.6 kg/cm^2^)	ASTM D648	°C	105 ± 2
Mold shrinkage	ASTM D955	%	1.5 ± 0.1

**Table 2:** Statistical analysis of the final Delphi technique.

No.	Item	M	Mo	SD	K–S Z-Test
1	PP extruder main screw output pressure control	4.54	5	0.499	1.941 **
2	PP extruder polymer temperature control	4.46	4	0.499	1.941 **
3	T-die lips adjustment depends on sheet thickness	4.38	4	0.487	2.219 **
4	Cooling rolls pressing stability	4.38	4	0.487	2.219 **
5	Cooling rolls temperature stability	4.31	4	0.462	2.496 **
6	Extruder main screw geometric design	4.23	4	0.421	2.774 **
7	Forming heating controller element stability	4.46	4	0.499	1.941 **
8	The deviation of forming heating constant temperature control area	4.23	4	0.421	2.774 **
9	Near to scenic sport or night markets	4.46	4	0.499	1.941 **
10	The maximum clamping force of thermoformer and mould forming area design	4.23	4	0.421	2.774 **

** *p* < 0.01.

**Table 3:** Calculation method for analysis of variance [21].

1. Degrees of freedom	*n*: level number dof=n−1
2. Variation	$V_{ar}=\frac{{SS}_{n}}{{DOF}_{n}}$
3. CF	$CF=\frac{{\left(\sum_{j=1}^{n}{SN}_{j}\right)}^{2}}{n}$
4. Total sum of squares	${SS}_{t}=\sum_{j=1}^{n}{\left({SN}_{j}\right)}^{2}-CF$
5. Error variation	${SS}_{error}={SS}_{total}-\sum {SS}_{n}$
6. *F* significance test	$F=\frac{V_{ar}}{V_{error}}$

**Table 4:** Control factor level table for PP-C90 lid manufacturing process.

Factor	Control Factor Description	Unit	Level 1	Level 2	Level 3
A	Polypropylene new material ratio	%	50	60	
B	Extrusion screw pressure speed	rpm	480	485	490
C	Polymer temperature	°C	210	220	230
D	T-die lips adjustment thickness	mm	0.53	0.56	0.59
E	Mirror wheel temperature stability	°C	25	30	35
F	Molding heating thermostatic control	°C	235	240	245
G	Molding vacuum pressure time	seconds	4.0	4.2	4.4
H	Forming mold area design	%	60	75	90

Note: The gray shaded part of Level 2 is set to the original design value.

**Table 5:** Numerical values for orthogonal array L_18_ and control factor levels A to H.

Exp.	A	B	C	D	E	F	G	H	P1	P2	P3	P4	P5	P6	P7
	%	rpm	°C	mm	°C	°C	sec	%							
**1**	50	480	210	0.51	25	235	4	60	3.50	3.40	3.35	3.30	3.30	3.30	3.25
**2**	50	480	220	0.53	30	240	4.2	75	3.50	3.60	3.45	3.45	3.40	3.40	3.45
**3**	50	480	230	0.55	40	245	4.4	90	3.50	3.30	3.60	3.35	3.45	3.35	3.40
**4**	50	485	210	0.51	30	240	4.4	90	3.50	3.40	3.30	3.65	3.45	3.40	3.40
**5**	50	485	220	0.53	40	245	4	60	3.50	3.45	3.40	3.35	3.50	3.40	3.45
**6**	50	485	230	0.55	25	235	4.2	75	3.50	3.30	3.30	3.20	3.25	3.50	3.40
**7**	50	490	210	0.53	25	245	4.2	90	3.50	3.30	3.25	3.30	3.30	3.30	3.60
**8**	50	490	220	0.55	30	235	4.4	60	3.50	3.50	3.50	3.50	3.50	3.40	3.35
**9**	50	490	230	0.51	40	240	4	75	3.50	3.40	3.30	3.30	3.35	3.35	3.30
**10**	60	480	210	0.55	40	240	4.2	60	3.50	3.30	3.30	3.35	3.45	3.40	3.30
**11**	60	480	220	0.51	25	245	4.4	75	3.30	3.30	3.40	3.30	3.35	3.35	3.30
**12**	60	480	230	0.53	30	235	4	90	3.35	3.40	3.30	3.45	3.35	3.40	3.30
**13**	60	485	210	0.53	40	235	4.4	75	3.30	3.25	3.35	3.40	3.30	3.30	3.35
**14**	60	485	220	0.55	25	240	4	90	3.30	3.35	3.40	3.35	3.30	3.25	3.35
**15**	60	485	230	0.51	30	245	4.2	60	3.30	3.40	3.30	3.25	3.30	3.30	3.30
**16**	60	490	210	0.55	30	245	4	75	3.35	3.35	3.25	3.35	3.30	3.30	3.30
**17**	60	490	220	0.51	40	235	4.2	90	3.45	3.40	3.25	3.30	3.40	3.40	3.35
**18**	60	490	230	0.53	25	240	4.4	60	3.35	3.30	3.25	3.30	3.35	3.45	3.50

**Table 6:** Factor response table for the S/N ratio in each experimental set.

	A	B	C	D	E	F	G	H
Level 1	32.6	33.7	33.6	35.1	33.1	33.9	36.4	34.5
Level 2	36.0	34.7	35.1	33.4	34.5	33.7	32.7	35.6
Level 3		34.4	34.1	34.4	35.3	35.3	33.7	32.7
E¹→²	3.4	1.0	1.5	−1.7	1.4	−0.2	−3.8	1.0
E²→³		−0.3	−1.1	1.0	0.8	1.6	1.1	−2.9
Range	3.4	1.0	1.5	1.7	2.2	1.6	3.8	2.9
Rank	2	8	7	5	4	6	1	3
Significant?	YES	NO	NO	YES	YES	NO	YES	YES

**Table 7:** Optimal combination of factor levels.

Factor	Control Factor Description	Unit	Level 1	Level 2	Level 3
A	Polypropylene new material ratio	%	50	60	
B	Extrusion screw pressure speed	rpm	480	485	490
C	Polymer temperature	°C	210	220	230
D	T-die lips adjustment thickness	mm	0.53	0.56	0.59
E	Mirror wheel temperature stability	°C	25	30	35
F	Molding heating thermostatic control	°C	235	240	245
G	Molding vacuum pressure time	seconds	4.0	4.2	4.4
H	Forming mold area design	%	60	75	90

Best factor level combination: A2, B2, C2, D1, E3, F3, G1, H2.

**Table 8:** Factor response table for quality characteristics in each experimental set.

	A	B	C	D	E	F	G	H
Level 1	3.39	3.37	3.35	3.351	3.34	3.36	3.35	3.37
Level 2	3.34	3.36	3.39	3.373	3.38	3.37	3.35	3.34
Level 3		3.36	3.35	3.363	3.37	3.35	3.38	3.37
E¹→²	−0.05	−0.02	0.03	0.021	0.05	0.01	0.00	−0.03
E²→³		0.00	−0.04	−0.010	−0.02	−0.02	0.03	0.03
Range	0.048	0.015	0.039	0.021	0.046	0.019	0.033	0.033
Rank	1	8	3	6	2	7	4	5
Significant?	YES	NO	YES	NO	YES	NO	YES	YES

**Table 9:** Preliminary analysis of variance data. "*" indicates significant and important control factors.

Factor	SS	DOF (n − 1)	Var	*F*-Value
A	0.0738	1	0.0738	13.4 *
B	0.0055	2	0.0028	0.5
C	0.0386	2	0.0193	3.5 *
D	0.0097	2	0.0048	0.9
E	0.0465	2	0.0232	4.2 *
F	0.0078	2	0.0039	0.7
G	0.0281	2	0.0141	2.5 *
H	0.0300	2	0.0150	2.7 *
Error	0.5971	108	0.0055	
Total	0.8484	125	0.0068	

**Table 10:** Consolidated error analysis of variance.

Factor	SS	DOF	Var	*F*-Value	Confidence	Significance *	Contribution
A	0.074	1	0.074	13.4	99.96%	Yes	8.70%
B	0.006	2	0.003	0.5	39.14%	No	0.65%
C	0.039	2	0.019	3.5	96.61%	Yes	4.55%
D	0.010	2	0.005	0.9	58.05%	No	1.14%
E	0.046	2	0.023	4.2	98.25%	Yes	5.48%
F	0.008	2	0.004	0.7	50.28%	No	0.92%
G	0.028	2	0.014	2.5	91.68%	Yes	3.32%
H	0.030	2	0.015	2.7	92.94%	Yes	3.54%
Others	0.011	2	0.006	1.0	63.44%	No	1.32%
Error	0.597	108	0.006	S = 7.44%			70.38%
Total	0.848	125	0.0068	* At least 90% confidence			100.00%

**Table 11:** Comparison of the original important factor design and optimal design model predictions.

Factor	Original Design		Optimal Design	
	Setting	Effect (dB)	Setting	Effect (dB)
A	A2	1.7	A2	1.7
B	(B2)		(B2)	
C	(C2)		(C3)	
D	D2	−0.9	D1	0.8
E	E2	0.2	E3	1.0
F	(F2)		(F3)	
G	G2	−1.6	G1	2.2
H	H2	1.3	H2	1.3
Average		34.3		34.3
Predicted by Additive Model		35.0		41.2

**Table 12:** Comparison of experimental confirmation values and optimal design-predicted values [38].

	*P1*	*P2*	*P3*	*P4*	*P5*	*P6*	*P7*	Ave.	*SD*	*S/N*	
										Experiment	Predicted
Original	3.35	3.50	3.45	3.35	3.40	3.40	3.35	3.41	0.06	34.9	35.0
	3.40	3.35	3.35	3.40	3.45	3.55	3.40				
Optimal	3.30	3.28	3.30	3.32	3.30	3.36	3.32	3.32	0.03	41.1	41.2
	3.28	3.34	3.30	3.34	3.32	3.38	3.34				
								Improvement =		6.21	6.24

**Table 13:** Explanation of research results on important key aspects of Delphi methods and optimal parameters of Taguchi method.

The Delphi Research Analysis Result		Taguchi Method Quality Control Research Results		
No.	The key factors( ten key subitems)	Factor	Description of important quality control factors	Optimal design parameters
1	PP extrusion main screw feed pressure control (revised: PP extrusion main screw discharge pressure control)	A	Polypropylene new material ratio	60%
2	PP extrusion resin temperature change control	B	Extrusion screw pressure speed	485 rpm
3	T-die lips adjustment sheet sta-bility (correction: die lips adjustment depends on sheet thickness)	C	Polymer temperature	220 °C
4	mirror wheel pressing stability	D	T-die lips adjustment thickness	0.53mm
5	mirror wheel temperature stability	E	Mirror wheel temperature stability	35 °C
6	extrusion driving screw geometric design	F	Molding heating thermostatic control	240 °C
7	molding heating con-troller element stability	G	Molding vacuum pressure time	4 sec
8	molding heating thermostatic control area error value	H	Forming mold area design	75%
9	molding vacuum and compressed air system stability	1. Factors A, D, E, G, H are used to reduce variation. 2. Factors C and F are used to adjust quality characteristics to target values. 3. Factor B can be used to reduce costs.		
10	molding machine maximum clamping force and molding area relation-ship design			

## Data Availability

Data are contained within the article.

## References

[1] Walther B.A., Yen N., Hu C.-S. (2021). Strategies, actions, and policies by Taiwan’s ENGOs, media, and government to reduce plastic use and marine plastic pollution. Mar. Policy.

[2] Silva A.L.P., Prata J.C., Walker T.R., Campos D., Duarte A.C., Soares A.M., Barcelò D., Rocha-Santos T. (2020). Rethinking and optimising plastic waste management under COVID-19 pandemic: Policy solutions based on redesign and reduction of single-use plastics and personal protective equipment. Sci. Total. Environ..

[3] Borrelle S.B., Ringma J., Law K.L., Monnahan C.C., Lebreton L., McGivern A., Murphy E., Jambeck J., Leonard G.H., Hilleary M.A. (2020). Predicted growth in plastic waste exceeds efforts to mitigate plastic pollution. Science.

[4] Clayton C.A.B. (2021). Building Collective Ownership of Single-Use Plastic Waste in Youth Communities: A Jamaican Case Study. Soc. Sci..

[5] Dybka-Stępień K., Antolak H., Kmiotek M., Piechota D., Koziróg A. (2021). Disposable food packaging and serving materials—Trends and biodegradability. Polymers.

[6] Jang Y., Kim K.N., Woo J. (2023). Post-consumer plastic packaging waste from online food delivery services in South Korea. Waste Manag..

[7] Clive M., Theresa C. (1998). Polypropylene: The Definitive User’s Guide and Databook.

[8] Somaye A. (2012). Polypropylene in the Industry of Food Packaging. https://www.intechopen.com/chapters/37229.

[9] Albert K.A., Cruz C.A., Palm J.P., Johnson R.W. (1985). Polypropylene resins for sheeting and thermoforming. J. Plast. Film Sheeting.

[10] Alotaibi M., Aldhafeeri T., Barry C. (2022). The Impact of Reprocessing with a Quad Screw Extruder on the Degradation of Polypropylene. Polymers.

[11] Daronde S., Kuthe A., Keerti S., Khatirkar R., Bagde A., Kamble M., Dahake S. (2022). The Effect of Vacuum on the Mechanical Properties of Sand Cast AA6061 Alloy. J. Mater. Eng. Perform..

[12] Kan M., Ipek O., Koru M. (2023). An investigation into the effect of vacuum conditions on the filling analysis of the pressure casting process. Int. J. Met..

[13] SUNWELL (2024). https://www.sunwellglobal.com.tw/.

[14] FCFC (2024). https://www.fcfc-plastics.com/pp-resin.htm/.

[15] Hos Win Enterprise Co., Ltd. (2024). https://www.laiwell.com/.

[16] Chen D.-C., Chen D.-F., Huang S.-M., Huang M.-J., Shyr W.-J., Chiou C.-F. (2021). Critical Success Factors to Improve the Business Performance of Tea Drink Chains. Sustainability.

[17] Jou Y.-T., Silitonga R.M., Lin M.-C., Sukwadi R., Rivaldo J. (2022). Application of Six Sigma Methodology in an Automotive Manufacturing Company: A Case Study. Sustainability.

[18] Abdulai M.N., Prah J., Walker E., Afrifa A.D. (2020). A fishbone analysis of the use of electronic health records (EHR) in a primary healthcare setting: The case of university of cape coast hospital. Int. J. Appl. Inf. Syst. (IJAIS).

[19] Chen D.-C., Chen D.-F., Huang S.-M., Shyr W.-J. (2022). The Investigation of Key Factors in Polypropylene Extrusion Molding Production Quality. Appl. Sci..

[20] Lakens D., Caldwell A.R. (2021). Simulation-based power analysis for factorial analysis of variance designs. Adv. Methods Prac. Psychol. Sci..

[21] Mustapha A.N., Zhang Y., Zhang Z., Ding Y., Yuan Q., Li Y. (2021). Taguchi and ANOVA analysis for the optimization of the microencapsulation of a volatile phase change material. J. Mater. Res. Technol..

[22] Fahmi N.K.A., Bintara R.D. (2022). Optimization Injection Molding Parameters of Polypropylene Materials to Minimize Flash Defects Using the Taguchi Method. Proceeding Int. Conf. Relig. Sci. Educ..

[23] Hiwa B., Ahmed Y.M., Rostam S. (2023). Evaluation of tensile properties of Meriz fiber reinforced epoxy composites using Taguchi method. Results Eng..

[24] Alim A.A., Roslan R., Nadzirah S., Saidi L.K., Menon P.S., Aziah I., Fu D.C., Sulaiman S.A., Murad N.A.A., Hamzah A.A. (2023). Geometrical Characterisation of TiO_2_-rGO Field-Effect Transistor as a Platform for Biosensing Applications. Micromachines.

[25] Patel N., Parihar P.L., Makwana J.S. (2023). Empirical study for Nusselt number optimization for the flow using ANOVA and Taguchi method. Case Stud. Therm. Eng..

[26] Lin K.-W., Chang Y.-C. (2021). Use of the Taguchi Method to Optimize an Immunodetection System for Quantitative Analysis of a Rapid Test. Diagnostics.

[27] Chen W.-H., Uribe M.C., Kwon E.E., Lin K.-Y.A., Park Y.-K., Ding L., Saw L.H. (2022). A comprehensive review of thermoelectric generation optimization by statistical approach: Taguchi method, analysis of variance (ANOVA), and response surface methodology (RSM). Renew. Sustain. Energy Rev..

[28] Abt G., Boreham C., Davison G., Jackson R., Nevill A., Wallace E., Williams M. (2020). Power, precision, and sample size estimation in sport and exercise science research. J. Sports Sci..

[29] Mohsin I., He K., Li Z., Zhang F., Du R. (2020). Optimization of the Polishing Efficiency and Torque by Using Taguchi Method and ANOVA in Robotic Polishing. Appl. Sci..

[30] Fei N.C., Mehat N.M., Kamaruddin S. (2022). Practical application of TAGUCHI optimization methodology to medical facilities: An integrated study. J. Mech. Med. Biol..

[31] Neag E., Stupar Z., Varaticeanu C., Senila M., Roman C. (2022). Optimization of Lipid Extraction from *Spirulina* spp. by Ultrasound Application and Mechanical Stirring Using the Taguchi Method of Experimental Design. Molecules.

[32] Oemar B., Chang W.-C. (2020). Taguchi method for optimizing process parameters in the production of activated carbon from rubber seed shell. Int. J. Adv. Manuf. Technol..

[33] Maguluri N., Suresh G., Rao K.V. (2023). Assessing the effect of FDM processing parameters on mechanical properties of PLA parts using Taguchi method. J. Thermoplast. Compos. Mater..

[34] Minh P.S., Dang H.-S., Ha N.C. (2023). Optimization of 3D Cooling Channels in Plastic Injection Molds by Taguchi-Integrated Principal Component Analysis (PCA). Polymers.

[35] Patnaik P.K., Mishra S.K., Swain P.T.R., Purohit A., Parija S.K., Panda S.S. (2022). Multi-Objective optimization and experimental analysis of Electro-Discharge Machining parameters via Gray-Taguchi, TOPSIS-Taguchi and PSI-Taguchi methods. Mater. Today Proc..

[36] Chalermthai B., Ashraf M.T., Bastidas-Oyanedel J.-R., Olsen B.D., Schmidt J.E., Taher H. (2020). Techno-Economic Assessment of Whey Protein-Based Plastic Production from a Co-Polymerization Process. Polymers.

[37] Huang W.-T., Tasi Z.-Y., Ho W.-H., Chou J.-H. (2022). Integrating Taguchi Method and Gray Relational Analysis for Auto Locks by Using Multiobjective Design in Computer-Aided Engineering. Polymers.

[38] Pazhamannil R.V., Hadidi H.M., Edacherian A., Puthumana G. (2023). Prediction of the mechanical properties of heat-treated fused filament fabrication thermoplastics using adaptive neuro-fuzzy inference system. J. Thermoplast. Compos. Mater..

